# Comprehensive Analysis of the PRDXs Family in Head and Neck Squamous Cell Carcinoma

**DOI:** 10.3389/fonc.2022.798483

**Published:** 2022-03-08

**Authors:** Ruoyan Cao, Weilin Zhang, Hongjian Zhang, Lixuan Wang, Xijuan Chen, Xianyue Ren, Bin Cheng, Juan Xia

**Affiliations:** ^1^ Hospital of Stomatology, Sun Yat-sen University, Guangzhou, China; ^2^ Guangdong Provincial Key Laboratory of Stomatology, Guangzhou, China; ^3^ Guanghua School of Stomatology, Sun Yat-sen University, Guangzhou, China

**Keywords:** HNSCC, peroxiredoxins, PRDX, prognosis, immune, bioinformatic analysis

## Abstract

The peroxidase family of peroxiredoxins (PRDXs) plays a vital role in maintaining the intracellular balance of ROS. However, their function in head and neck squamous cell carcinoma (HNSCC) has not been investigated. We therefore explored the value of PRDXs in HNSCC. We found that the expression of PRDX1, PRDX4, and PRDX5 in HNSCC increased while the expression of PRDX2 decreased. Moreover, the high expression of PRDX4/5/6 indicated a poor prognosis. Lower expression of PRDX1/5 was linked to more immune cell infiltration, higher expression of immune-related molecules and a more likely response to anti-PD-1 treatment. Moreover, PRDX5 knockdown inhibited HNSCC cell proliferation, invasion and metastasis and it might promote apoptosis through its antioxidant property. Taken together, our study highlights the potential role of PRDXs in HNSCC. The function of PRDX5 in the development of HNSCC and the formation of the immune microenvironment makes it a promising potential therapeutic target.

## Introduction

Head and neck squamous cell carcinoma (HNSCC) is the sixth most frequent tumor, annually accounting for ~ 830 000 new cases and 430 000 deaths worldwide (1). Despite significant advancements in therapeutic strategies, HNSCC remains a significant health problem as it presents a 5-year overall survival rate of 50% (2). Therefore, more information on the molecular mechanisms responsible for the progress of HNSCC should be obtained to develop more novel therapeutic targets.

The peroxiredoxin (PRDX) family of peroxidases comprises six members (PRDX1 to PRDX6) that constitute a broad range of cellular antioxidant defenders. Based on the number of cysteines, PRDXs are divided into three categories: typical 2-cysteine peroxiredoxins (PRDX1–PRDX4), atypical 2-cysteine peroxiredoxins (PRDX5), and 1-cysteine peroxiredoxins (PRDX6). Although these proteins have different functions in antioxidant protection and cellular redox regulation, they share a common molecular mechanism. PRDXs catalyze the reduction of H2O2, peroxynitrite, and organic hydroperoxides to maintain the intracellular balance of reactive oxygen species (ROS) (3, 4). ROS imbalance is closely associated with tumorigenesis and progression. Moderate ROS generation stimulates cell proliferation and differentiation; however, excessive ROS induces oxidative damage to proteins, lipids, and DNA and ultimately leads to cell structure damage and death (5). Thus, the PRDX family has gradually gained attention in the field of cancer research.

According to recent reports, the PRDX family is dysregulated in multiple tumors. PRDX1 is up-regulated in some tumors such as pancreatic ductal adenocarcinoma, non-small cell lung cancer, cervical cancer and breast cancer, and promotes the growth and metastasis of cancer cells (6–9). PRDX2 facilitates the proliferation of colorectal cancer cells and non-small cell lung cancer cells (10, 11). PRDX3 and PRDX5, two mitochondrial PRDXs, are associated with unfavorable survival in endometrial cancer and ovarian cancer (12, 13). PRDX4 is a metastasis-related marker in oral squamous cell carcinoma, and high PRDX5 expression correlates with a poor prognosis (14). PRDX6 plays a crucial role in mitochondrial dysfunction and ferroptosis (15–17). However, the role of PRDXs in the occurrence and progression of HNSCC has not yet been elucidated.

Thus, in this study, we focused on the biological roles of the PRDX family in HNSCC. In our results, PRDX5 and PRDX4 were upregulated in HNSCC patients and were associated with a shorter survival time. Patients that showed higher PRDX1/5 expression also had lower immune cell infiltration and lower expression of immune-related molecules. Knocking down PRDX5 *in vitro* inhibited the proliferation, migration, and invasion of HNSCC and increased apoptosis, which might result from ROS elevation. In addition, patients with low expression of PRDX1/5 was more likely to respond to the PD-1 inhibitor.

## Material and Methods

### Data Source

Level 3 RNASeq, miRNASeq data and the corresponding clinical information of the HNSCC datasets were obtained from The Cancer Genome Atlas database (TCGA) (https://portal.gdc.cancer.gov/). RNASeq data was transformed into transcripts per kilobase million (TPM) values.

### Gene Set Variation Analysis and Functional State Signatures

We divided TCGA HNSCC cohort into different groups (PRDXs-high and PRDXs-low) based on the “survminer” R package. To understand the biological pathways in which PRDXs could be involved, we performed Gene Set Variation Analysis (GSVA) using “GSVA” R packages. The gene sets of “hallmark” was downloaded from the “msigdbr” R package. An adjusted P-value of less than 0.01 was considered significant. In addition, CancerSEA (http://biocc.hrbmu.edu.cn/CancerSEA/home.jsp) was used to evaluate the relationship between PRDXs and 14 functional states based on the GSE103322 data set, including angiogenesis, apoptosis, cell cycle, differentiation, DNA damage, DNA repair, EMT, hypoxia, inflammation, invasion, metastasis, proliferation, quiescence, and stemness.

### Tumor Immunology Analysis

We obtained tumor purity *via* ESTIMATE algorithm (https://bioinformatics.mdanderson.org/estimate/). We quantified the immune cell infiltration using single-sample gene set enrichment analysis (ssGSEA) based on 23 types of immune cells signatures (18). The differences of immune-related molecules were also assessed in the different groups of PRDXs.

### Methylation, Copy Number Variation and lncRNA Analysis of PRDX in HNSCC

Methylation and copy number variation (CNV) of the PRDXs in HNSCC datasets was assessed and visualized based on MEXPRESS (https://mexpress.be/). The relationship between lncRNA and PRDXs was assessed using spearman correlation analysis. |Correlation coefficient| ≥ 0.3 and adjusted P-value < 0.05 were defined as significant.

### Immunotherapy Response Prediction

To indirectly predict the immunotherapy response of different expression of PRDX1/5/6, we assessed the similarity of gene expression profiles between PRDX1/5/6 and immunotherapy- treated melanoma patients based on subclass mapping (19, 20).

### Cell Transfection

A small interfering RNA (siRNA) targeting PRDX5 (si-PRDX5) and a negative control siRNA (si-NC) were designed and purchased from GenePharma (Suzhou, China). The sequence of si-PRDX5#1 was 5’- GGUGGCCUGUCUGAGUGUUdTdT-3’, and the sequence of si-PRDX5#2 was 5’- GCCUGGCACCCAAUAUCAUdTdT-3’. Lipofectamine 3000 (Invitrogen, Carlsbad, CA, United States) was used to transfect si-PRDX5 and si-NC into HNSCC cells following the instructions of the manufacturer.

### Quantitative Real-Time PCR

Total RNA was isolated from NOK, HSC2, UM1, HN6, HSC4, and SCC4 cells by the RNA-quick purification kit (ESscience Biotech, China) following the manufacturer’s instructions. A total of 2 µg RNA was synthesized from cDNA using HiScript III-RT SuperMix (Vazyme, China). The obtained cDNA product was used for real-time quantification PCR (RT-qPCR). The following primers were used: PRDX5, forward 5′-CTCCTGGCTGATCCCACT-3′, and reverse 5′- CACTATGCCATCCTGTACCAC-3′; GAPDH, forward 5′-CTCCTCCTGTTCGACAGTCAGC-3′, and reverse 5′ -CCCAATACGACCAAATCCGTT-3.

### Western Blotting

Total proteins of HNSCC cell lines were extracted with RIPA lysis buffer and subjected to 10% sodium dodecyl sulfate-polyacrylamide gel electrophoresis (SDS-PAGE). The proteins were transferred to a 0.22-µm PVDF membrane (Millipore, United States) and blocked with 5% non-fat skimmed milk at RT for 1 h. Next, the membranes were incubated with primary antibodies against PRDX5 (Abclonal, China) and GAPDH (Proteintech, China) at 4°C overnight. After that, the membranes were incubated with species-specific secondary antibodies at RT for 1 hour. Finally, the bands were detected using the enhanced chemiluminescence system (Bio-Rad, United States).

### Cell Counting Kit-8 (CCK-8) Assay

HN6 and HSC4 cells with transfected siRNAs were seeded at 1500 cells/200 µl per well in 96-well plates. CCK-8 kit (Beyotime, China) was applied to assess cell viability at different time points according to the protocol of the manufacturer.

### Colony Formation Assay

HN6 and HSC4 cells were transfected with siRNAs, seeded in 6-well plates (500 cells per well), and cultured for 14 days. Then, colonies were fixed by methanol and stained with 0.5% crystal violet solution, and visible colonies were manually counted.

### Wound Healing Assay

HN6 and HSC4 cells transfected with siRNAs were grown in 6-well plates and cultured at 37 °C. When the cells grew to 90% confluence, a 200 µl tip was used to create a thin scratch. The supernatant cells were washed with PBS. Subsequently, the cells were subjected to a serum-free medium for starvation. The area of the scratch was recorded at 0 h and 24 h with an inverted microscope (ZEISS, Germany).

### Transwell Assay

Cell invasion assays were performed in transwell chambers (8 µm pores, Corning, United States) precoated with Matrigel (BD biosciences, United States). In detail, a total of 200 µl serum-free medium containing 8 × 105 cells were added in the upper chambers, and 600 µl medium containing 20% FBS was placed in the lower chambers. After 24 h of incubation, the invading cells were fixed with 4% paraformaldehyde for 30 minutes, stained with crystal violet for 1 h, and counted under an inverted microscope (ZEISS, Germany).

### Apoptosis Assay

Apoptosis of HN6 and HSC4 cells was detected by Muse Annexin V & Dead Cell Assay Kit (KeyGEN, China) according to the manufacture protocol. HN6 and HSC4 cells transfected with siRNAs were starved in serum-free medium and resuspended with Annexin V buffer. Then, the cells were incubated with Annexin V-FITC and PI in the dark. A flow cytometer (CytoFlex) was used to assess apoptotic cells.

### Mitochondrial ROS (mtROS) Measurement

We employed MitoSOX Red reagent (Thermo Fisher, USA) to determinate mtROS according to the manufacturer’s instructions. HN6 and HSC4 cells transfected with siRNA were incubated with 5 µM MitoSox Red dye at 37°C for 10 minutes and then washed with PBS three times. The fluorescence signal of the labeled samples was analyzed *via* a flow cytometer (CytoFlex).

## Results

### High Expression of PRDX4 and PRDX5 Predicts HNSCC Prognosis

**Figure 1:**
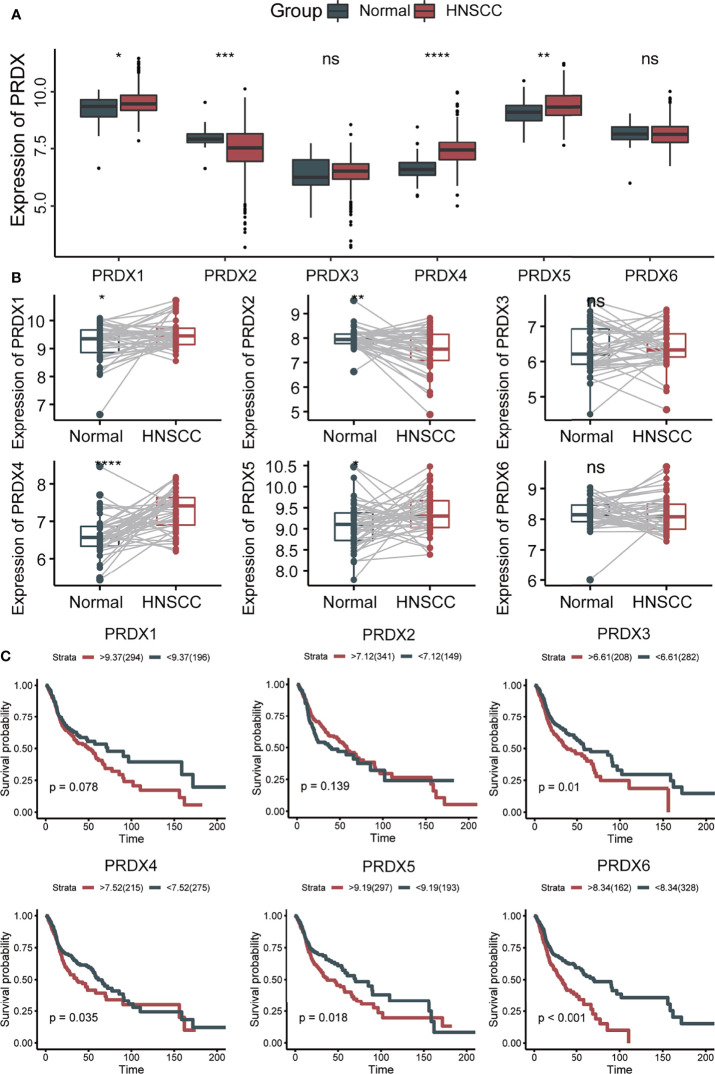
The expression and prognosis of PRDXs in HNSCC. **(A)** Box plot of PRDXs expression between normal tissues and HNSCC tissues. **(B)** Box plot of PRDXs expression between normal tissues and paired HNSCC tissues. **(C)** Survival curve of PRDXs in HNSCC. ns, not significant; *p-value < 0.05; **p-value < 0.01; ***p-value < 0.001; ****p-value < 0.0001.

First, we analyzed the expression levels of PRDXs (PRDX1-6) between HNSCC and normal controls in the TCGA database. Compared to the normal tissues, PRDX1, PRDX4 and PRDX5 were significantly upregulated, while PRDX2 was significantly downregulated in HNSCC tumors (**Figure 1A**). Similar results were found in paired HNSCC and normal tissues (**Figure 1B**). Kaplan-Meier survival analysis indicated that higher PRDX3, PRDX4, PRDX5 and PRDX6 expression levels were correlated with poor prognosis (**Figure 1C**). Additionally, we found that PRDX3/4/5/6 could be an independent risk factor for the survival of HNSCC (**Table 1**). High expression of PRDX3/4/5/6 had a significantly higher mortality.

**Table 1:** Relationship between PRDXs and overall survival of HNSCC.

Outcome	Crude Model		Model I		Model II	
	HR (95%)	*P*-value	HR (95%)	*P*-value	HR (95%)	*P*-value
PRDX1						
Low expression	Reference		Reference		Reference	
High expression	1.29 (0.97, 1.70)	0.079	1.33 (1.00, 1.76)	0.051	1.27 (0.95, 1.69)	0.101
PRDX2						
Low expression	Reference		Reference		Reference	
High expression	0.81 (0.61, 1.07)	0.140	0.81 (0.61, 1.07)	0.141	0.79 (0.59, 1.05)	0.109
PRDX3						
Low expression	Reference		Reference		Reference	
High expression	1.43 (1.09, 1.88)	0.010	1.46 (1.11, 1.91)	0.007	1.40 (1.05, 1.85)	0.020
PRDX4						
Low expression	Reference		Reference		Reference	
High expression	1.34 (1.02, 1.75)	0.036	1.35 (1.03, 1.77)	0.032	1.37 (1.04, 1.81)	0.026
PRDX5						
Low expression	Reference		Reference		Reference	
High expression	1.41 (1.06, 1.87)	0.019	1.42 (1.07, 1.89)	0.016	1.44 (1.08, 1.93)	0.012
PRDX6						
Low expression	Reference		Reference		Reference	
High expression	1.81 (1.37, 2.40)	<0.0001	1.87 (1.41, 2.48)	<0.0001	1.88 (1.42, 2.50)	<0.0001

Model I adjusted for age and sex.

Model II adjusted for age, sex, alcohol history, hpv status, grade and stage.

### Gene Set Variation Analysis and Functional State Signatures

**Figure 2:**
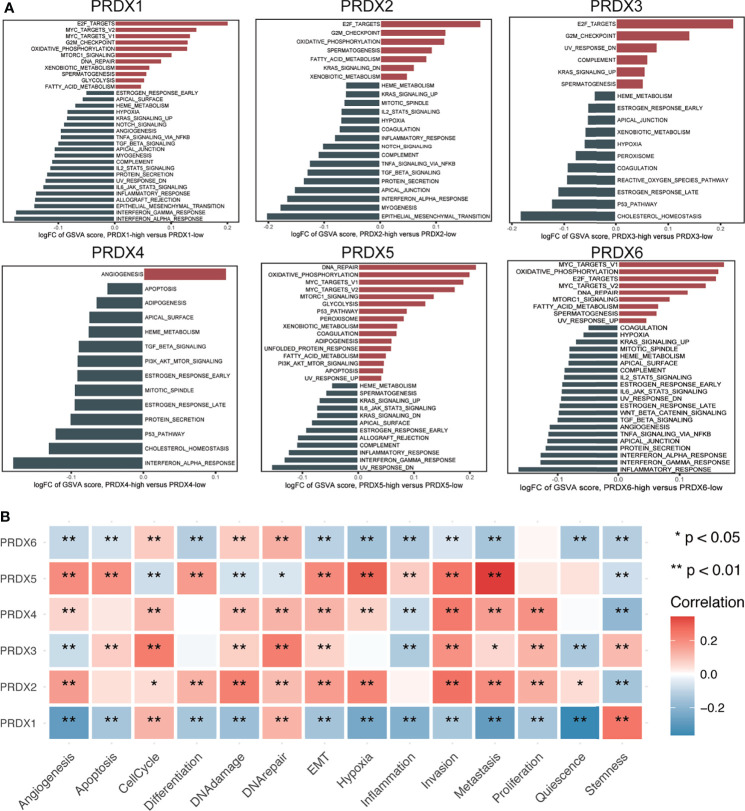
Function analysis of PRDXs in HNSCC. **(A)** GSVA analysis of hallmark gene set in PRDXs. **(B)** The relationship between PRDXs and functional states. *p-value < 0.05; **p-value < 0.01.

GSVA analysis was performed to explore the possible roles of PRDXs in HNSCC patients. PRDX1, PRDX5 and PRDX6 were positively associated with DNA repair, MYC targets V1/V2, mTORC1 signaling, oxidative phosphorylation and fatty acid metabolism. Similarity, PRDX1, PRDX5 and PRDX6 were negatively associated with heme metabolism, KRAS signaling up, IL6_JAK_STAT3 signaling, complement, interferon gamma response and inflammatory response. PRDX2 and PRDX3 were positively associated with E2F targets, G2M checkpoint and spermatogenesis. PRDX3 and PRDX4 were negatively associated with heme metabolism, estrogen response, p53 pathway and cholesterol homeostasis (**Figure 2A**).

Next, a single cell transcriptome cohort (GSE103322) was used to assess the relationship between PRDXs and 14 functional state signatures in HNSCC tumor cells based on the CancerSEA database. PRDX1 was positively correlated with cell cycle, DNA repair and stemness while PRDX1 was negatively correlated with angiogenesis, apoptosis, differentiation, DNA damage, EMT, hypoxia, inflammation, invasion, metastasis, proliferation and quiescence. PRDX2 was positively correlated with angiogenesis, cell cycle, differentiation, DNA damage, DNA repair, EMT, hypoxia, invasion, metastasis, proliferation and quiescence, while it was negatively correlated with stemness. PRDX3 was positively correlated with apoptosis, cell cycle, differentiation, DNA damage, DNA repair, invasion, metastasis, proliferation and stemness, while it was negatively correlated with angiogenesis, inflammation and quiescence. PRDX4 was positively correlated with angiogenesis, cell cycle, DNA damage, DNA repair, EMT, hypoxia, invasion, metastasis and proliferation, while it was negatively correlated with inflammation and stemness. PRDX5 was positively correlated with angiogenesis, apoptosis, differentiation, EMT, hypoxia, inflammation, invasion and metastasis, while it was negatively correlated with cell cycle, DNA damage, DNA repair and stemness. PRDX6 was positively correlated with cell cycle, DNA damage and DNA repair, while it was negatively correlated with angiogenesis, apoptosis, differentiation, EMT, hypoxia, inflammation, invasion and metastasis, quiescence and stemness (**Figure 2B**).

### The Relationship Between Immune Microenvironment and PRDXs

**Figure 3:**
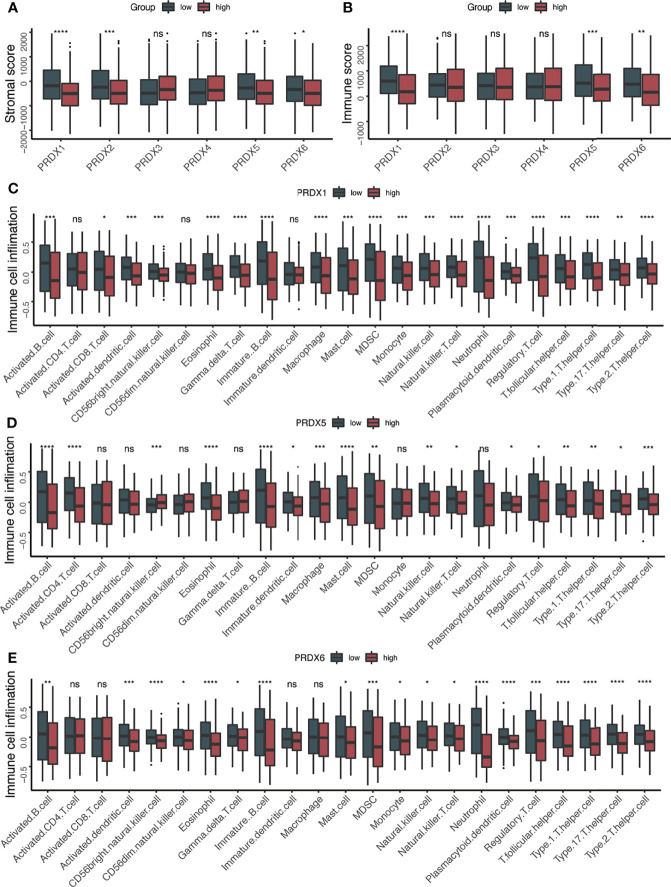
Higher immune cell infiltration in PRDX1/5 low group. **(A)** The box plots of stromal score between PRDXs high and PRDXs low patients. **(B)** The box plots of immune score between PRDXs high and PRDXs low patients. **(C–E)** The box plots of immune cell infiltration between PRDX1/5/6 high and PRDX1/5/6 low patients. ns, not significant; *p-value < 0.05; **p-value < 0.01; ***p-value < 0.001; ****p-value < 0.0001.

**Figure 4:**
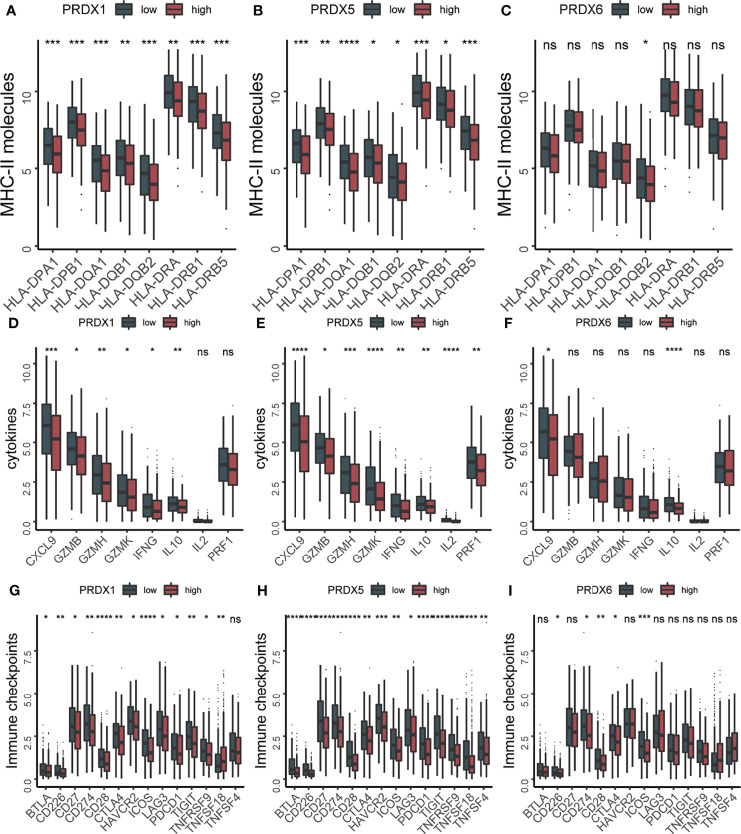
The expression levels of immune related molecules were increased in PRDX1/5 low patients. **(A–C)** The box plots of MHC-II molecules between PRDX1/5/6 high and PRDX1/5/6 low patients. **(D–F)** The box plots of cytokines between PRDX1/5/6 high and PRDX1/5/6 low patients. **(G–I)** The box plots of immune checkpoints between PRDX1/5/6 high and PRDX1/5/6 low patients. ns, not significant; *p-value < 0.05; **p-value < 0.01; ***p-value < 0.001; ****p-value < 0.0001.

Considering that both PRDXs were associated with the immune-related pathways, the relationship between the immune microenvironment and PRDXs was analyzed. Patients with low PRDX1/5/6 expression showed higher stromal and immune scores than PRDX1/5/6-high patients (**Figures 3A, B**). PRDX2-low patients gained more stromal score, while a higher immune score was not observed in the PRDX2-low patients. Thus, we further explored the difference in tumor microenvironment between PRDX1/5/6-low and PRDX1/56-high. Consistently with the higher immune score in the PRDX1/5/6-low group, we found increased immune cell infiltration in the PRDX1/5/6-low group (**Figures 3C–E**). Additionally, many immune-related genes were up-regulated in PRDX1/5-low patients, including MHC-II molecules (HLA-DRB5, HLA-DPB1, HLA-DRA, HLA-DRB1, HLA-DQA1, HLA-DQB2, HLA-DPA1 and HLA-DQB1), cytotoxic effectors (GZMB, GZMH and GZMK), cytokines (CXCL9, IFNG and IL10) and immune checkpoints (BTLA, CD226, CD27, CD274, CD28, CTLA4, HAVCR2, ICOS, LAG3, PDCD1, TIGIT, TNFRSF9 and TNFSF18) (**Figures 4A–I**).

### Methylation, CNV and lncRNA Analysis of PRDX in HNSCC

**Figure 5:**
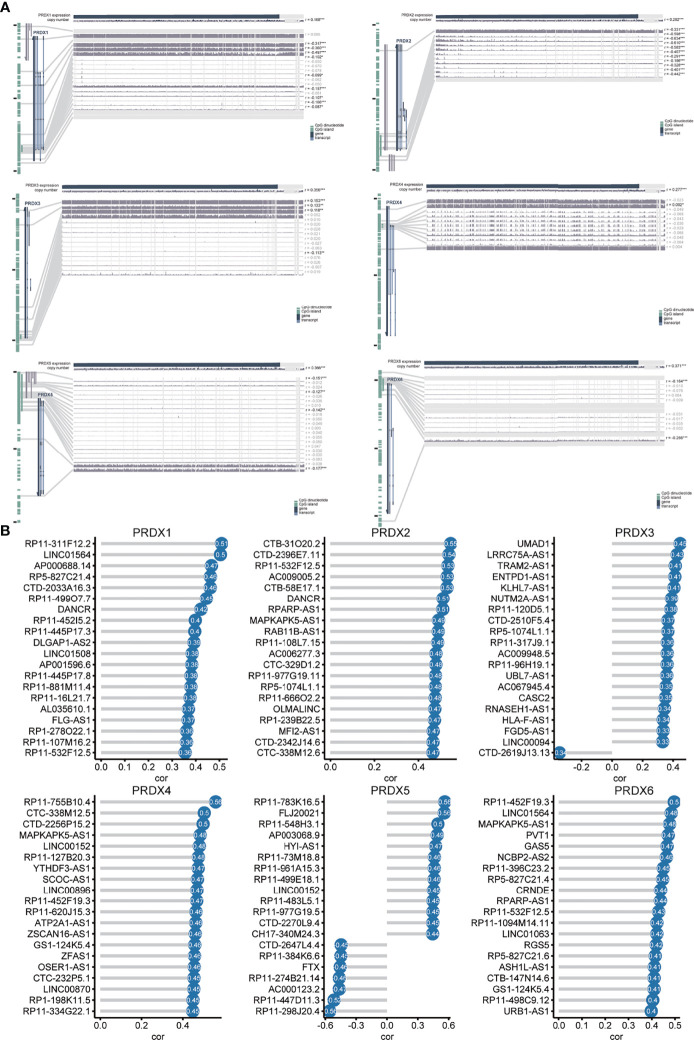
Methylation, CNV and lncRNA analysis of PRDX in HNSCC **(A)** The correlation of PRDXs with methylation and CNVs. **(B)** The correlation of PRDXs with lncRNAs.

To explore the mechanisms regulating the expression of PRDXs in HNSCC, we assessed the relationship between PRDXs and methylation, CNV and lncRNA. PRDX1 was negatively correlated with 9 methylation probes (from r = -0.087 to r = -0.317). PRDX2 showed a relatively strong correlation with methylation (from r = -0.186 to r =-0.610). PRDX3 was positively or negatively associated with methylation probes. PRDX5 was negatively correlated with 4 methylation probes (from r = -0.127 to r = -0.177). PRDX6 was also negatively associated with methylation. Only one methylation probe was positively associated with PRDX4. The expression of PRDXs was significantly associated with CNV, especially PRDX6 (r = 0.371) and PRDX5 (r = 0.366) (**Figure 5A**). In addition, the correlation between lncRNA and PRDXs was shown in **Figure 5B** and **Table S1**.

### PRDX1/PRDX5-Low Patients Are More Sensitive to Immune Therapy

**Figure 6:**
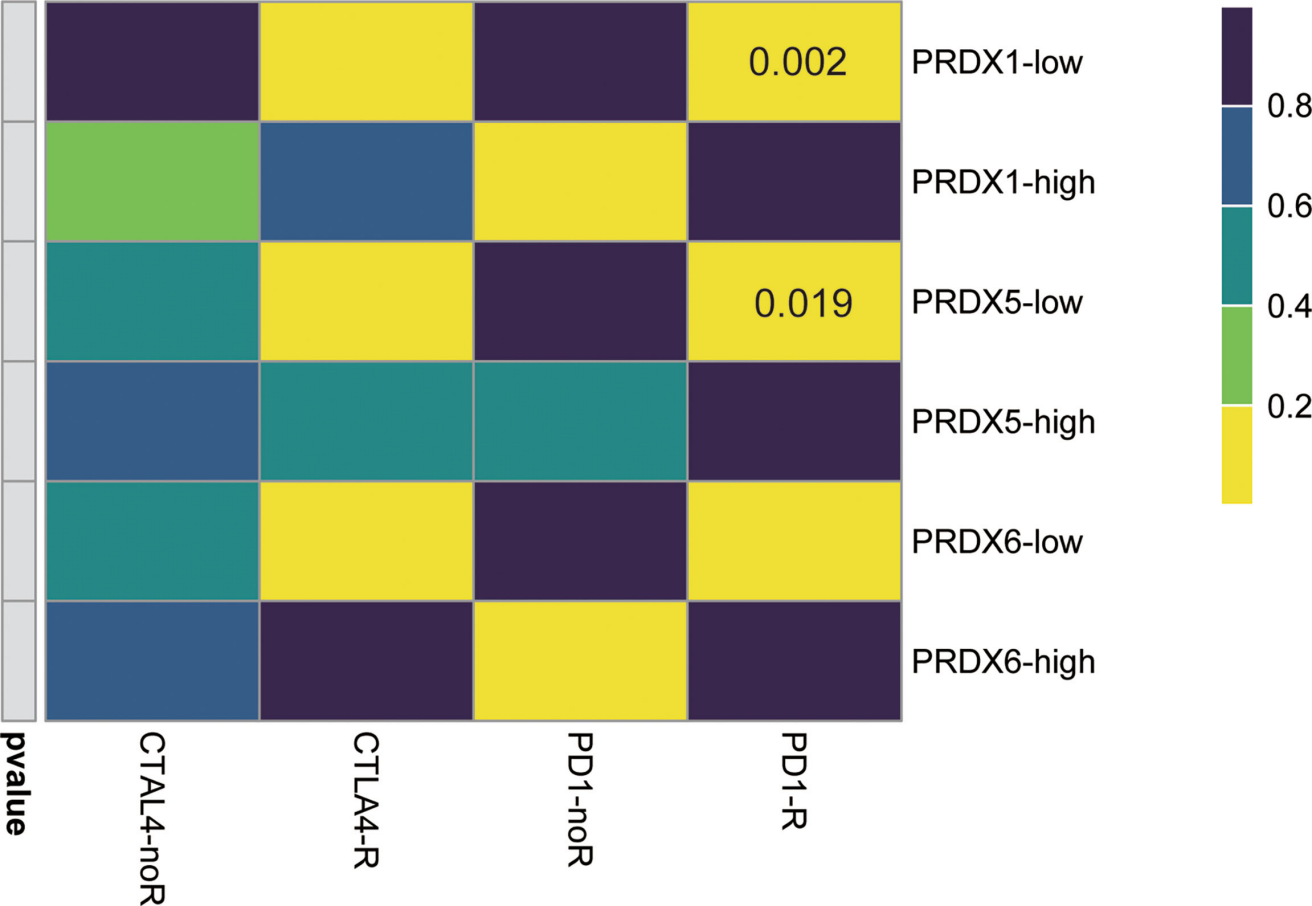
Immunotherapeutic response of PRDX1/5/6. PRDX1/5 low patients may be more likely response to the PD-1 inhibitor by SubMap analysis in HNSCC.

Because of the negative correlation between PRDX1/5/6 and immune score, we further explored the response of different PRDX1/5/6 expression to immunotherapy. The results indicated that PRDX1/5-low was more likely to respond to anti-PD-1 (**Figure 6**).

### Silencing PRDX5 Inhibits HNSCC Cell Proliferation, Migration, and Invasion and Induces Cell Apoptosis

**Figure 7:**
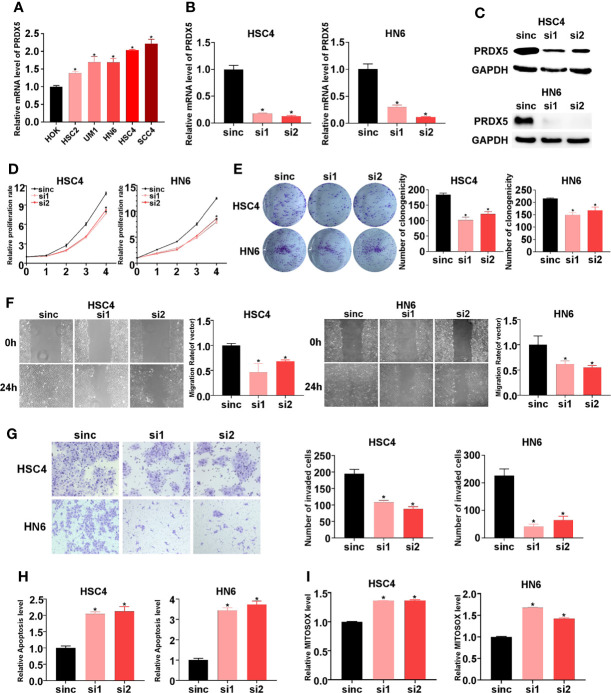
Impact of PRDX5 knockdown on biological behavior of HNSCC cells *in vitro*. **(A)** The mRNAs levels of PRDX5 in HOK and multiple HNSCC cells. **(B, C)** The mRNA and protein levels of PRDX5 in HSC4 and HN6 cell transfected with PRDX5 siRNA. **(D)** Relative proliferation rate of HSC4 and HN6 cell transfected with PRDX5 siRNA. **(E)** Colony formation capacity of HSC4 and HN6 cell transfected with PRDX5 siRNA **(F)** Migration ability was measured using a wound healing assay in HSC4 and HN6 cell transfected with PRDX5 siRNA. **(G)** Invasion abilities were detected by a Transwell assay with Matrigel. **(H)** The effect of PRDX5 knockdown on HSC4 and HN6 cell apoptosis. **(I)** Relative mtROS level HSC4 and HN6 cell transfected with PRDX5 siRNA. *p-value < 0.05.

PRDX4 has been reported to promote progression of oral squamous cell carcinoma (14); however, the biological role of PRDX5 in HNSCC is unknown. Firstly, we compared the mRNA levels of PRDX5 in a human normal epithelial cell line (NOK) and in HNSCC cell lines. qPCR confirmed that PRDX5 was upregulated in multiple HNSCC cell lines (**Figure 7A**). To elaborate on the biological function of PRDX5, PRDX5 was knocked down using siRNAs in HSC4 and HN6 cell lines (**Figures 7B, C**). CCK8 and colony formation assays were used to test the effect of PRDX5 on the proliferation of HNSCC. Silencing PRDX5 in HSC4 and HN6 cells reduced cell viability and colony numbers (**Figures 7D, E**). The wound-healing assay showed that silencing PRDX5 significantly decreased cell migration (**Figure 7F**). Consistently, the transwell invasion assay showed that the number of cells in the siRNA group that passed through the Matrigel membrane was lower than that of the NC group (**Figure 7G**), suggesting decreased invasion. Meanwhile, PRDX5 knockdown provoked apoptosis of HNSCC cells (**Figure 7H**). Furthermore, we found that silencing PRDX5 increased mtROS levels in HNSCC cells (**Figure 7I**). Thus, these results demonstrated that PRDX5 could promote HNSCC progression through its antioxidant property.

## Discussion

Rapidly growing cancerous cells need to maintain high levels of anabolism by ingesting large amounts of nutrients, leading to excessive ROS production and subsequent oxidative stress. To avoid oxidative stress, the expression of antioxidant enzyme systems, such as PRDXs, is tightly regulated (21, 22). Although PRDXs has been analyzed in pan-cancer level, the detailed role of PRDXs in prognosis, expression-regulatory relationships, functional state signatures, immune microenvironment and the response to immune treatment in HNSCC is lacking. Thus, we further explored the effect of PRDXs family in HNSCC. In this study, we found that the expression of PRDX1/4/5 increased in HNSCC patients while the expression of PRDX2 decreased. Higher expression of PRDX4/5/6 was correlated with a poor prognosis. In addition, low expression of PRDX1/5 tended to be more likely to response to anti-PD-1 treatment.

PRDX2 was associated with prognosis in multiple cancers, such as colorectal cancer (11), hepatocellular carcinoma (23) and esophageal squamous cell carcinoma (24). Though PRDX2 did not predict HNSCC prognosis, we still found that PRDX2 was positively associated with aggressive phenotype, such as angiogenesis, EMT, invasion, metastasis and proliferation, which was in line with oral squamous cell carcinoma (OSCC). Overexpression of PRDX2 could promote OSCC cell proliferation, cell-cycle progression, migration and inhibit apoptosis (25). PRDX3 acts as an oncogene in hepatocellular carcinoma (26), prostate cancer (27) and renal cell carcinoma (28). Knockdown of PRDX3 is associated with decreased ATP synthase and cellular ATP levels, resulting in slower cell growth (26). In our study, we also found that PRDX3 might invoke HNSCC proliferation.

PRDX4 is the only member of the PRDXs family located in the endoplasmic reticulum, and its expression is upregulated in multiple tumors, including OSCC, lung cancer, prostate cancer, colorectal cancer, ovarian cancer and breast cancer (29). We also found that the upregulated PRDX4 might play an oncogene function in provoking proliferation, metastasis, and invasion, leading to a poor prognosis of HNSCC patients, which is confirmed in OSCC (14). The silencing of PRDX4 is related to the generation of ROS in the endoplasmic reticulum, which diffuses to the nucleus and induces DNA damage (30). In addition, PRDX4 can reduce the expression of adhesion molecules (31), thereby increasing the possibility of metastasis and invasion. These may partially explain the involvement of PRDX4 in the proliferation, metastasis and invasion of HNSCC.

PRDX6 is the only 1-cysteine PRDX located in the cytoplasm and translocates to acidic organelles upon its phosphorylation (32). Loss of PRDX6 leads to mitochondrial dysfunction, low growth rate, decreased migration and invasion abilities and ferroptosis (1, 15, 16, 33, 34). In our study, patients with higher PRDX6 expression had shorter survival times, suggesting that PRDX6 might act as an oncogene in HNSCC. However, we also found that PRDX6 was negatively correlated with angiogenesis, EMT, invasion and metastasis. Thus, the function of PRDX6 in HNSCC needs to be further explored.

PRDX5 is the only atypical 2-Cys PRDXs, mainly located in mitochondrion. PRDX5 is elevated and drives tumorigenic phenotype in colon cancer (35), non-small cell lung cancer (36), and gastric cancer (37). In our study, we also found that PRDX5 could act as an oncogene in HNSCC. Knocking down of PRDX5 significantly increased mitochondrial ROS levels, promoted apoptosis and suppressed proliferation, migration and invasion of HNSCC cells. The relationship between excess ROS and aggressive phenotypes has been demonstrated in multiple tumors (22).

Owing to the relationship between ROS and immune, we also explore the connection between immune microenvironment and PRDXs. Compared with PRDX1/5-high patients, we found an increase in increased immune cell infiltration, HLA molecules, chemokines, cytolytic activity-related genes and immune checkpoint molecules in PRDX1/5-low patients, indicating that PRDX1/5-low exhibited a hot tumor state. Several possible mechanisms may contribute to this phenotype. For one thing, in the PRDX1/5-low group, a high level of ROS can induce the production of chemokines (for example, CXCL9), thereby recruiting T cells into the tumor (38). For another thing, high ROS levels can lead to cellular oxidative damage and immunogenic cell death in the tumor, providing a potential antigenic stimulation for the immune system, and further attract immune cells into the tumor (39). Additionally, tumor-specific MHC-II expression is correlated with the activation of IFNG pathway, higher levels of Th1 cytokines, and CD4^+^/CD8^+^ lymphocytes infiltration (40). Therefore, it is reasonable to find that patients with low PRDX1/5 have a more promising response to anti-PD-1 therapy. Furthermore, PRDX5 actives the Nrf2 signaling pathway, which could protect tumor cells from anti-tumor immunity (36, 41). Therefore, our results highlight the value of targeting PRDX1/5 to inhibit the progression of HNSCC and turn “cold” tumors into “hot” tumors.

Although this study systematically analyzed the value of PRDXs and verified the function of PRDX5 in HNSCC, some limitations are acknowledged. The PRDXs expression and survival analysis is mainly based on the data retrieved from the TCGA database, and therefore multi-centered cohorts are required to confirm the results presented here. In addition, it is necessary to elucidate the in-depth mechanism by which PRDX5 promotes the aggressive phenotype of HNSCC.

## Conclusion

In summary, we found that PRDX1/2/4/5 were dysregulated in HNSCC and that higher expression of PRDX4/5/6 was associated with a shorter survival time. The possible function of PRDX5 in the development of HNSCC and the regulation of the immune microenvironment makes this protein a promising therapeutic target.

## Data Availability Statement

The original contributions presented in the study are included in the article/**Supplementary Material**. Further inquiries can be directed to the corresponding authors.

## Author Contributions

JX and BC designed the study. RC performed all the bioinformatics analysis described here. WZ performed *in vitro* experiments. RC, HZ, and WZ wrote and edited the manuscript. LW, XC, and XR collected and examined the data. JX and BC supervised the project. All authors read and approved the final manuscript.

## Funding

This study was supported by the National Natural Science Foundation of China (No. 81870769, 81700979), Guangdong Financial fund for High-Caliber Hospital Construction (174-2018-XMZC-0001-03-0125/D-05).

## Conflict of Interest

The authors declare that the research was conducted in the absence of any commercial or financial relationships that could be construed as a potential conflict of interest.

## Publisher’s Note

All claims expressed in this article are solely those of the authors and do not necessarily represent those of their affiliated organizations, or those of the publisher, the editors and the reviewers. Any product that may be evaluated in this article, or claim that may be made by its manufacturer, is not guaranteed or endorsed by the publisher.

## Supplementary Material

Supplementary Table 1 The correlation of PRDXs and lncRNAs.

## References

[1] Bray F Ferlay J Soerjomataram I Siegel RL Torre LA Jemal A. Global Cancer Statistics 2018: GLOBOCAN Estimates of Incidence and Mortality Worldwide for 36 Cancers in 185 Countries. CA: Cancer J Clin (2018) 68:394–424. doi: 10.3322/caac.21492 30207593

[2] Leemans CR Braakhuis BJM Brakenhoff RH. The Molecular Biology of Head and Neck Cancer. Nat Rev Cancer (2011) 11:9–22. doi: 10.1038/nrc2982 21160525

[3] Castaldo SA Freitas JR Conchinha NV Madureira PA. The Tumorigenic Roles of the Cellular REDOX Regulatory Systems. Oxid Med Cell Longevity (2016) 2016:8413032. doi: 10.1155/2016/8413032 PMC4670861 26682014

[4] Chen Y Cong Y Quan B Lan T Chu X Ye Z. Chemoproteomic Profiling of Targets of Lipid-Derived Electrophiles by Bioorthogonal Aminooxy Probe. Redox Biol (2017) 12:712–8. doi: 10.1016/j.redox.2017.04.001 PMC5390668 28411555

[5] Zhao C Cao W Zheng H Xiao Z Hu J Yang L. Acid-Responsive Nanoparticles as a Novel Oxidative Stress-Inducing Anticancer Therapeutic Agent for Colon Cancer. Int J Nanomed (2019) 14:1597–618. doi: 10.2147/IJN.S189923 PMC6400122 30880968

[6] Dahou H Minati MA Jacquemin P Assi M. Genetic Inactivation of Peroxiredoxin-I Impairs the Growth of Human Pancreatic Cancer Cells. Antioxid (Basel Switzerland) (2021) 10:570. doi: 10.3390/antiox10040570 PMC8068151 33917763

[7] Lu E Hu X Pan C Chen J Xu Y Zhu X. Up-Regulation of Peroxiredoxin-1 Promotes Cell Proliferation and Metastasis and Inhibits Apoptosis in Cervical Cancer. J Cancer (2020) 11:1170–81. doi: 10.7150/jca.37147 PMC6959069 31956363

[8] O’Leary PC Terrile M Bajor M Gaj P Hennessy BT Mills GB. Peroxiredoxin-1 Protects Estrogen Receptor α From Oxidative Stress-Induced Suppression and Is a Protein Biomarker of Favorable Prognosis in Breast Cancer. Breast Cancer Res: BCR (2014) 16:R79. doi: 10.1186/bcr3691 25011585 PMC4226972

[9] Song C Xiong G Yang S Wei X Ye X Huang W. PRDX1 Stimulates Non-Small-Cell Lung Carcinoma to Proliferate *via* the Wnt/β-Catenin Signaling. Panminerva Med (2020). doi: 10.23736/S0031-0808.20.03978-6 32881473

[10] Chen Y Yang S Zhou H Su D. PRDX2 Promotes the Proliferation and Metastasis of Non-Small Cell Lung Cancer *In Vitro* and *In Vivo* . BioMed Res Int (2020) 2020:8359860. doi: 10.1155/2020/8359860 32908916 PMC7474358

[11] Wang W Wei J Zhang H Zheng X Zhou H Luo Y. PRDX2 Promotes the Proliferation of Colorectal Cancer Cells by Increasing the Ubiquitinated Degradation of P53. Cell Death Dis (2021) 12:605. doi: 10.1038/s41419-021-03888-1 34117220 PMC8196203

[12] Byun JM Kim SS Kim KT Kang MS Jeong DH Lee DS. Overexpression of Peroxiredoxin-3 and -5 Is a Potential Biomarker for Prognosis in Endometrial Cancer. Oncol Lett (2018) 15:5111–8. doi: 10.3892/ol.2018.7909 PMC5835875 29541251

[13] Zhang Y Lin S Chen Y Yang F Liu S. LDH-Apromotes Epithelial-Mesenchymal Transition by Upregulating ZEB2 in Intestinal-Type Gastric Cancer. OncoTargets Ther (2018) 11:2363–73. doi: 10.2147/OTT.S163570 PMC5931238 29740212

[14] Chang K-P Yu J-S Chien K-Y Lee C-W Liang Y Liao C-T. Identification of PRDX4 and P4HA2 as Metastasis-Associated Proteins in Oral Cavity Squamous Cell Carcinoma by Comparative Tissue Proteomics of Microdissected Specimens Using iTRAQ Technology. J Proteome Res (2011) 10:4935–47. doi: 10.1021/pr200311p 21859152

[15] López-Grueso MJ Lagal DJ García-Jiménez ÁF Tarradas RM Carmona-Hidalgo B Peinado J. Knockout of PRDX6 Induces Mitochondrial Dysfunction and Cell Cycle Arrest at G2/M in HepG2 Hepatocarcinoma Cells. Redox Biol (2020) 37:101737. doi: 10.1016/j.redox.2020.101737 33035814 PMC7554216

[16] Lu B Chen XB Hong YC Zhu H He QJ Yang B. Identification of PRDX6 as a Regulator of Ferroptosis. Acta Pharmacol Sin (2019) 40:1334–42. doi: 10.1038/s41401-019-0233-9 PMC6786318 31036877

[17] Ma S Zhang X Zheng L Li Z Zhao X Lai W. Peroxiredoxin 6 Is a Crucial Factor in the Initial Step of Mitochondrial Clearance and Is Upstream of the PINK1-Parkin Pathway. Antioxid Redox Signaling (2016) 24:486–501. doi: 10.1089/ars.2015.6336 26560306

[18] Zhang B Wu Q Li B Wang D Wang L Zhou YL. m(6)A Regulator-Mediated Methylation Modification Patterns and Tumor Microenvironment Infiltration Characterization in Gastric Cancer. Mol Cancer (2020) 19:53. doi: 10.1186/s12943-020-01170-0 32164750 PMC7066851

[19] Hoshida Y Brunet JP Tamayo P Golub TR Mesirov JP. Subclass Mapping: Identifying Common Subtypes in Independent Disease Data Sets. PloS One (2007) 2:e1195. doi: 10.1371/journal.pone.0001195 18030330 PMC2065909

[20] Roh W Chen PL Reuben A Spencer CN Prieto PA Miller JP. Integrated Molecular Analysis of Tumor Biopsies on Sequential CTLA-4 and PD-1 Blockade Reveals Markers of Response and Resistance. Sci Trans Med (2017) 9:379. doi: 10.1126/scitranslmed.aah3560 PMC5819607 28251903

[21] Forshaw TE Holmila R Nelson KJ Lewis JE Kemp ML Tsang AW. Peroxiredoxins in Cancer and Response to Radiation Therapies. Antioxid (Basel Switzerland) (2019) 8:11. doi: 10.3390/antiox8010011 PMC6356878 30609657

[22] Ismail T Kim Y Lee H Lee DS Lee HS. Interplay Between Mitochondrial Peroxiredoxins and ROS in Cancer Development and Progression. Int J Mol Sci (2019) 20:4407. doi: 10.3390/ijms20184407 PMC6770548 31500275

[23] Xu M Xu J Zhu D Su R Zhuang B Xu R. Expression and Prognostic Roles of PRDXs Gene Family in Hepatocellular Carcinoma. J Trans Med (2021) 19:126. doi: 10.1186/s12967-021-02792-8 PMC7995729 33771165

[24] Feng AL Han X Meng X Chen Z Li Q Shu W. PRDX2 Plays an Oncogenic Role in Esophageal Squamous Cell Carcinoma *via* Wnt/β-Catenin and AKT Pathways. Clin Trans Oncol: Off Publ Fed Spanish Oncol Societies Natl Cancer Institute Mexico (2020) 22:1838–48. doi: 10.1007/s12094-020-02323-9 32130676

[25] Chuerduangphui J Ekalaksananan T Heawchaiyaphum C Vatanasapt P Pientong C. Peroxiredoxin 2 Is Highly Expressed in Human Oral Squamous Cell Carcinoma Cells and Is Upregulated by Human Papillomavirus Oncoproteins and Arecoline, Promoting Proliferation. PloS One (2020) 15:e0242465. doi: 10.1371/journal.pone.0242465 33332365 PMC7746188

[26] Liu Z Hu Y Liang H Sun Z Feng S Deng H. Silencing PRDX3 Inhibits Growth and Promotes Invasion and Extracellular Matrix Degradation in Hepatocellular Carcinoma Cells. J Proteome Res (2016) 15:1506–14. doi: 10.1021/acs.jproteome.5b01125 26983019

[27] Whitaker HC Patel D Howat WJ Warren AY Kay JD Sangan T. Peroxiredoxin-3 Is Overexpressed in Prostate Cancer and Promotes Cancer Cell Survival by Protecting Cells From Oxidative Stress. Br J Cancer (2013) 109:983–93. doi: 10.1038/bjc.2013.396 PMC3749568 23880827

[28] Yu R Yao J Ren Y. A Novel circRNA, Circnup98, a Potential Biomarker, Acted as an Oncogene *via* the miR-567/PRDX3 Axis in Renal Cell Carcinoma. J Cell Mol Med (2020) 24:10177–88. doi: 10.1111/jcmm.15629 PMC7520319 32729669

[29] Jia W Chen P Cheng Y. PRDX4 and Its Roles in Various Cancers. Technol Cancer Res Treat (2019) 18:1533033819864313. doi: 10.1177/1533033819864313 31311441 PMC6636222

[30] Jain P Dvorkin-Gheva A Mollen E Malbeteau L Xie M Jessa F. NOX4 Links Metabolic Regulation in Pancreatic Cancer to Endoplasmic Reticulum Redox Vulnerability and Dependence on PRDX4. Sci Adv (2021) 7:eabf7114. doi: 10.1126/sciadv.abf7114 33962950 PMC8104867

[31] Guo X Noguchi H Ishii N Homma T Hamada T Hiraki T. The Association of Peroxiredoxin 4 With the Initiation and Progression of Hepatocellular Carcinoma. Antioxid Redox Signaling (2019) 30:1271–84. doi: 10.1089/ars.2017.7426 29687726

[32] Sorokina EM Feinstein SI Zhou S Fisher AB. Intracellular Targeting of Peroxiredoxin 6 to Lysosomal Organelles Requires MAPK Activity and Binding to 14-3-3ϵ. Am J Physiol Cell Physiol (2011) 300:C1430–41. doi: 10.1152/ajpcell.00285.2010 PMC3118628 21346153

[33] He Y Xu W Xiao Y Pan L Chen G Tang Y. Overexpression of Peroxiredoxin 6 (PRDX6) Promotes the Aggressive Phenotypes of Esophageal Squamous Cell Carcinoma. J Cancer (2018) 9:3939–49. doi: 10.7150/jca.26041 PMC6218759 30410598

[34] Hu X Lu E Pan C Xu Y Zhu X. Overexpression and Biological Function of PRDX6 in Human Cervical Cancer. J Cancer (2020) 11:2390–400. doi: 10.7150/jca.39892 PMC7066013 32201510

[35] Ahn HM Yoo JW Lee S Lee HJ Lee HS Lee DS. Peroxiredoxin 5 Promotes the Epithelial-Mesenchymal Transition in Colon Cancer. Biochem Biophys Res Commun (2017) 487:580–6. doi: 10.1016/j.bbrc.2017.04.094 28431931

[36] Cao X Chen XM Xiao WZ Li B Zhang B Wu Q. ROS-mediated Hypomethylation of PRDX5 Promotes STAT3 Binding and Activates the Nrf2 Signaling Pathway in NSCLC. Int J Mol Med (2021) 47:573–82. doi: 10.3892/ijmm.2020.4819 PMC7797423 33416106

[37] Kim B Kim YS Ahn HM Lee HJ Jung MK Jeong HY. Peroxiredoxin 5 Overexpression Enhances Tumorigenicity and Correlates With Poor Prognosis in Gastric Cancer. Int J Oncol (2017) 51:298–306. doi: 10.3892/ijo.2017.4013 28535004

[38] Uzhachenko RV Bharti V Ouyang Z Blevins A Mont S Saleh N. Metabolic Modulation by CDK4/6 Inhibitor Promotes Chemokine-Mediated Recruitment of T Cells Into Mammary Tumors. Cell Rep (2021) 35:108944. doi: 10.1016/j.celrep.2021.108944 33826903 PMC8383195

[39] Mao D Hu F Yi Z Kenry, Xu S Yan S Luo Z. AIEgen-Coupled Upconversion Nanoparticles Eradicate Solid Tumors Through Dual-Mode ROS Activation. Sci Adv (2020) 6:eabb2712. doi: 10.1126/sciadv.abb2712 32637621 PMC7319755

[40] Axelrod ML Cook RS Johnson DB Balko JM. Biological Consequences of MHC-II Expression by Tumor Cells in Cancer Clinical Cancer Research: An Official Journal of the American Association for Cancer Research. Clin Cancer Res: Official J American Assoc Cancer Res (2019) 25:2392–402. doi: 10.1158/1078-0432.CCR-18-3200 PMC6467754 30463850

[41] Kitamura H Onodera Y Murakami S Suzuki T Motohashi H. IL-11 Contribution to Tumorigenesis in an NRF2 Addiction Cancer Model. Oncogene (2017) 36:6315–24. doi: 10.1038/onc.2017.236 28714957

